# Enzyme Inhibition by Bioactive Compounds from Olive (*Olea europaea* L.) and Pomegranate (*Punica granatum* L.): Systematic Review of In Vitro Studies

**DOI:** 10.3390/molecules31122134

**Published:** 2026-06-17

**Authors:** Robert Vučina, Doris Drmač, Valentina Rezić, Dušan Čulum, Martin Kondža

**Affiliations:** 1 Faculty of Pharmacy, University of Mostar, Matice hrvatske bb, 88000 Mostar, Bosnia and Herzegovina; robertvucina7@gmail.com (R.V.); doris.drmac@farf.sum.ba (D.D.); valentina.rezic@farf.sum.ba (V.R.); 2 Faculty of Science, University of Sarajevo, Zmaja od Bosne 33-35, 71000 Sarajevo, Bosnia and Herzegovina; dusan.culum@pmf.unsa.ba; 3 Faculty of Food Technology Osijek, Josip Juraj Strossmayer University of Osijek, Franje Kuhača 18, 31000 Osijek, Croatia

**Keywords:** *Olea europaea* L., *Punica granatum* L., enzyme inhibition, bioactive compounds, in vitro, polyphenols, drug metabolism

## Abstract

Compounds from olive (*Olea europaea* L.) and pomegranate *(Punica granatum* L.) have many beneficial effects on human health. This review paper considers the inhibitory potential, under in vitro conditions, of bioactive components of olive and pomegranate on different enzyme systems. Research shows that olive polyphenols (oleuropein, hydroxytyrosol, luteolin, and oleocanthal), as well as pomegranate polyphenols (punicalagin, urolithin A, ellagic acid), inhibit cyclooxygenase and lipoxygenase enzymes, which are associated with inflammatory processes. They also show an inhibitory effect on acetylcholinesterase, butyrylcholinesterase, and β-secretase, which opens up the possibility of a strong neuroprotective effect. Olive and pomegranate polyphenols also have an inhibitory effect on enzymes involved in carbohydrate metabolism, such as amylase and glucosidase, and can help fight diabetes and regulate human metabolism. In addition, polyphenols and extracts of both plants showed an inhibitory effect on cytochrome P450 enzymes, which metabolize most drugs. These data open up the possibility of interactions with certain groups of drugs. The current evidence supports the view that olive and pomegranate polyphenols act as biologically versatile compounds with considerable pharmaceutical and nutraceutical potential. Future investigations integrating enzymology, metabolomics, molecular docking, and clinical validation will be essential for translating these promising in vitro findings into evidence-based therapeutic applications.

## 1. Introduction

**Figure 1:**
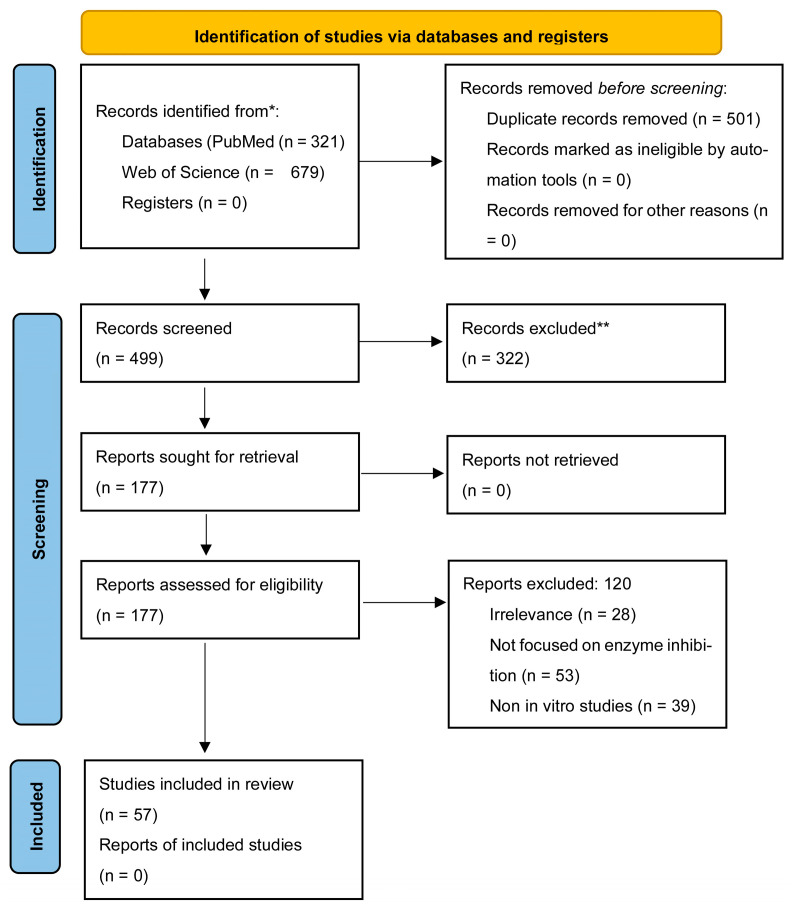
PRISMA identifications for this study. * Records were identified exclusively through PubMed and Web of Science databases. Scopus was not included in the search strategy because PubMed and Web of Science were considered sufficient to capture the biomedical and pharmacological literature relevant to the review topic. ** Records excluded after title and abstract screening due to irrelevance, lack of focus on enzyme inhibition, different plant species, or non–in vitro study design.

There is a lot of research showing that polyphenols of plant origin are of exceptional value to human health. They exhibit anti-inflammatory, antioxidative, anti-mutagenic, and anti-allergic effects [1,2]. Olive products have been used for centuries for their health benefits, and olive oil is one of the foundations of the Mediterranean diet that has proven cardioprotective properties as well as other beneficial effects on human health. Olive oil is rich in polyphenols, among which the most notable are oleuropein, hydroxytyrosol, and oleocanthal; these have strong antioxidant, anti-tumor, and anti-inflammatory properties. In addition to olive oil, olive leaves and other by-products of olive cultivation have been shown to be rich sources of bioactive compounds. Oleuropein is particularly noteworthy, as it has antihypertensive, hypoglycemic, antioxidant, and hypocholesterolemic effects [3,4,5,6]. No less important for human health is the pomegranate, which is also considered a medicinal Mediterranean plant. Like the olive, the pomegranate has often been used in traditional medicine to treat various health problems, most often diseases of the digestive tract, such as diarrhea or ulcers. The pomegranate has shown many pharmacological activities, some of these include anti-inflammatory, anti-tumor, anti-bacterial, anti-malarial, anti-fungal, and anti-diabetic effects. Pomegranate extracts are extremely rich in bioactive components, such as proanthocyanidins, tannins, important minerals, organic acids, flavonoids, vitamin C, terpenes, and many more. Some of the most important polyphenols found in pomegranate are punicalagin, punicalin, and gallagic acid, which have shown anti-cancer effects [7,8,9]. The pomegranate has proven to be particularly effective in the prevention and treatment of metabolic syndrome. Various parts of the pomegranate (arils, peels, seeds, and flowers) have been investigated, and it has been proven that pomegranate can be beneficial in reducing blood pressure, body weight, total cholesterol, and low-density lipoprotein cholesterol [10,11]. Polyphenols from both plants have shown excellent neuroprotective potential, especially in the prevention of Alzheimer’s disease, by modulating pathological changes and eliminating harmful molecules [12,13,14]. The aim of this work was to provide a detailed overview of the currently available knowledge on how the bioactive components of pomegranate and olive inhibit certain enzymes associated with chronic diseases, as well as the possible interactions they may have with drugs, using the Preferred Reporting Items for Systematic Reviews and Meta-Analyses (Figure 1) [15].

**Figure 2:**
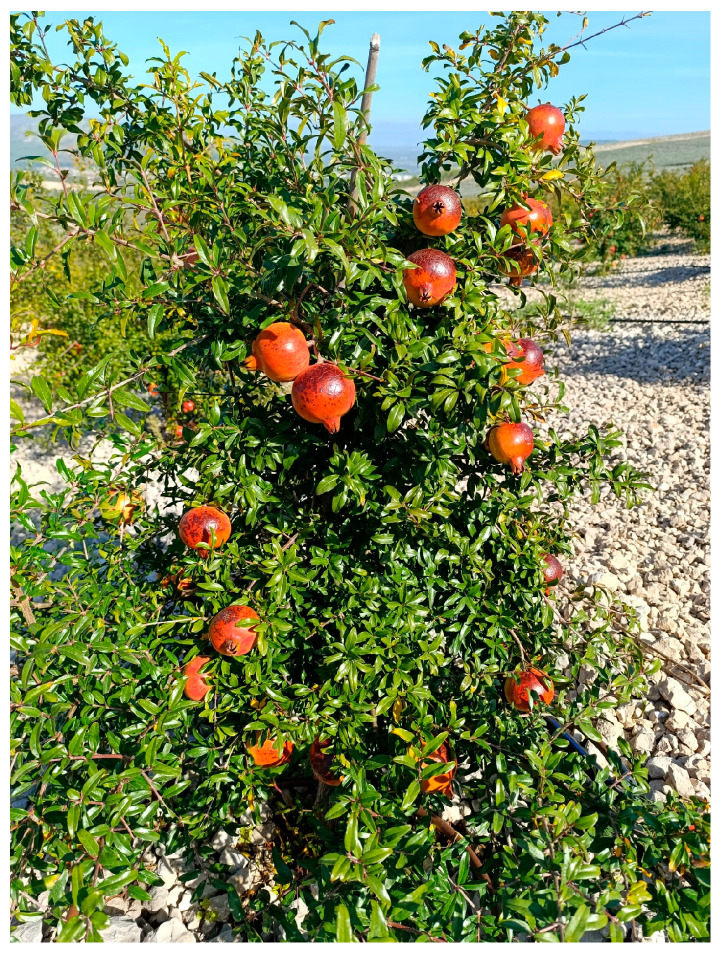
*Punica granatum* L.

**Figure 3:**
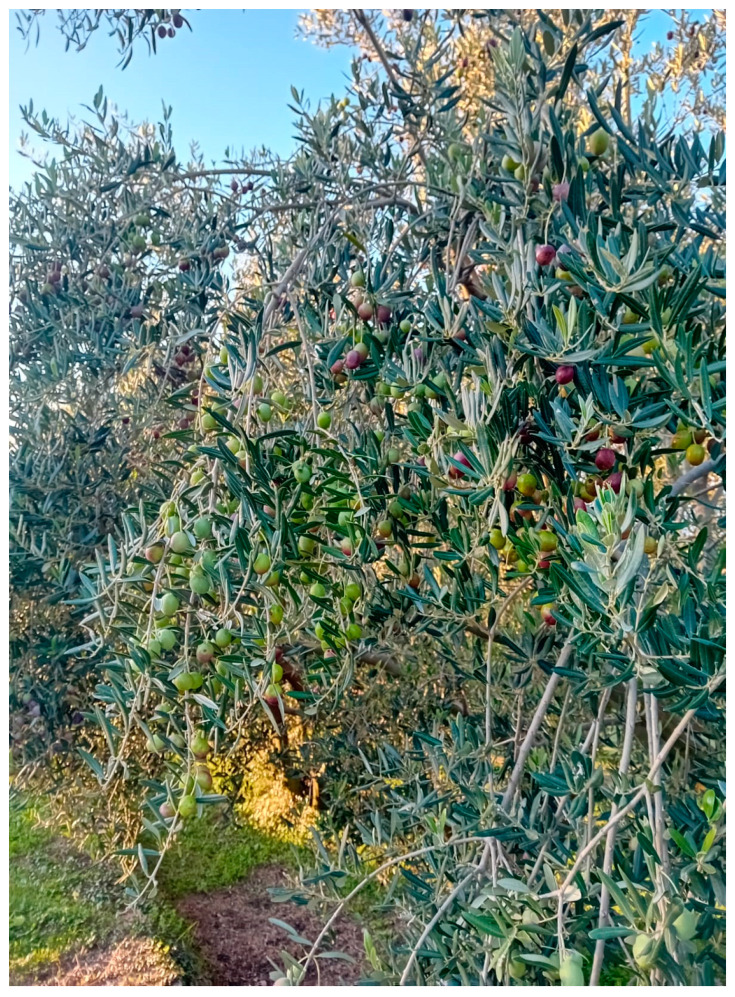
*Olea europaea* L.

We considered the inhibition of enzymes associated with inflammation, specifically the inhibition of cyclooxygenase (COX) and lipoxygenase (LOX) enzymes [15]. We also considered two enzymes associated with type 2 diabetes, α-glucosidase and α-amylase, whose inhibition is associated with the suppression of postprandial glucose peaks [16]. In view of potential anti-Alzheimer’s effects, we considered the inhibition of acetylcholinesterase (AChE), butyrylcholinesterase (BChE), and β-secretase (BACE-1), enzymes associated with the pathology of Alzheimer’s disease (AD) [17,18,19]. We also considered a possible inhibitory effect on cytochrome P450 (CYP) enzymes that could lead to changes in the metabolism of certain drugs [20] (Figure 2 and Figure 3).

## 2. Inhibition of Enzymes Mediating Inflammatory Pathways

Phenolic compounds from *Olea europaea* L., especially oleocanthal, show strong anti-inflammatory activity by directly inhibiting COX enzymes. In in vitro studies, oleocanthal was found to block both COX-1 and COX-2, which leads to reduced production of prostaglandins, key molecules responsible for pain and inflammation [21]. Interestingly, this effect is very similar to that of common anti-inflammatory drugs such as ibuprofen, suggesting that olive oil naturally contains compounds with drug-like activity [22]. Additional studies confirm that this inhibition is dose-dependent and helps reduce chronic inflammation [23,24]. Beyond simple enzyme inhibition, oleocanthal has been shown to interfere with the conversion of arachidonic acid into pro-inflammatory mediators, which places it directly within the central inflammatory pathway [22]. This mechanism explains why regular consumption of olive oil is associated with lower inflammatory marker levels across various studies [24]. Even in the small quantities found in food, oleocanthal shows a significant ability to inhibit processes, indicating its importance in biological functions. Dual inhibition of COX-1 and COX-2 is crucial, as targeting just one isoform can cause adverse effects, whereas inhibiting both offers a more balanced anti-inflammatory action [22]. Overall, these findings support the idea that olive phenolics are not only antioxidants but also active modulators of inflammatory enzyme systems [24].

Polyphenols from *Punica granatum* L. also show strong effects on the COX pathway. Cell-based in vitro investigations revealed that pomegranate juice and its principal constituents, including punicalagin, markedly suppressed the expression of COX-2, an enzyme primarily linked to disease-related inflammation [25]. As a result, the production of prostaglandins decreases, leading to a lower inflammatory response. In addition, studies on pomegranate metabolites, such as urolithin A, show reduced COX-2 activity and prostaglandin levels, suggesting that both the original compounds and their metabolites contribute to this effect [26]. Reviews also confirm that pomegranate polyphenols play an important role in controlling inflammatory enzymes [27,28]. These effects are particularly relevant because COX-2 is often overexpressed in chronic inflammatory diseases and cancer, making it an important therapeutic target [25]. Pomegranate compounds not only reduce enzyme expression but also interfere with signaling processes that lead to COX activation, enhancing their overall effectiveness [26]. The presence of multiple polyphenols allows for a broader and more stable inhibition of COX activity compared to single-compound interventions [27]. Furthermore, pomegranate extracts have shown consistent results across different experimental models, supporting their reliability as anti-inflammatory agents [28]. Altogether, these findings highlight the strong potential of pomegranate as a natural regulator of COX-mediated inflammation [25].

Olive phenolics also affect another important inflammatory pathway controlled by LOX. In vitro studies demonstrate that hydroxytyrosol and oleuropein are capable of inhibiting 5-LOX activity in immune cells, leading to lower production of leukotriene B4, a molecule known to foster inflammation and attract immune cells to damaged sites [29]. This means that olive compounds can reduce inflammation not only through COX inhibition but also by limiting leukotriene formation. Supporting this dual impact, reviews demonstrate that olive phenolics are active in both the COX and LOX pathways [24,30]. The inhibition of LOX is particularly important because leukotrienes play a major role in chronic inflammatory diseases, including asthma and arthritis [30]. Hydroxytyrosol, a potent olive phenolic, demonstrates superior effectiveness in inhibiting LOX activity when contrasted with other substances [29]. In addition, these phenolics can reduce oxidative stress, which is closely linked to LOX activation, further enhancing their anti-inflammatory potential [24]. The combined inhibition of COX and LOX pathways suggests that olive phenolics can control inflammation at multiple levels within the same biochemical cascade [30]. This multi-target effect is considered highly beneficial because it reduces the likelihood of compensatory activation of alternative inflammatory pathways [24].

Pomegranate compounds also influence the LOX pathway, although this effect is less directly studied compared to COX. Available studies suggest that pomegranate polyphenols can reduce the formation of leukotrienes, indicating inhibition of LOX-related processes [31]. In addition, pomegranate has strong antioxidant activity, which helps reduce oxidative stress, an important trigger of LOX activation. This indirect effect further supports the role of pomegranate in controlling inflammation through multiple pathways [27,32]. The antioxidant capacity of pomegranate is largely attributed to its high content of ellagitannins and flavonoids, which help neutralize reactive oxygen species involved in inflammatory signaling [32]. By reducing oxidative stress, these compounds can limit the activation of LOX enzymes and subsequent leukotriene production [27]. Some studies also suggest that pomegranate extracts may interfere with upstream processes that lead to LOX activation, providing an additional level of control [31]. The combined direct and indirect effects make pomegranate particularly effective in modulating inflammation [32]. This supports the idea that pomegranate acts as a multi-functional anti-inflammatory agent targeting several steps of the inflammatory cascade [27] (Table 1).

## 3. Inhibition of Enzymes Regulating Carbohydrate Digestion

The aqueous olive leaf extract (*Olea europaea* L., UOLE) demonstrated strong inhibitory activity against α-amylase in vitro. The extract exhibited a low IC_50_ value of 0.75 µg/mL, indicating high inhibitory potency compared to the standard antidiabetic drug acarbose (IC_50_ = 15.74 µg/mL). Kinetic analysis suggested a mixed-type inhibition mechanism, as reflected by changes in both V_max_ and K_m_, indicating interaction with both the free enzyme and the enzyme–substrate complex. This activity is likely attributed to synergistic interactions between olive leaf polysaccharides and phenolic compounds, including flavonoids, which may interact with α-amylase through hydrogen bonding and hydrophobic interactions. These findings suggest that olive leaf extract represents a potent natural inhibitor of carbohydrate-digesting enzymes with potential relevance for type 2 diabetes management [33]. This pronounced inhibitory effect of UOLE was further supported by Mansour et al., who reported an identical IC_50_ value of 0.75 µg/mL for α-amylase inhibition in vitro. When compared with other plant-derived inhibitors, such as polysaccharides from *Allium roseum* (IC_50_ = 100 µg/mL), UOLE demonstrated substantially higher activity, supporting its potential role in modulating carbohydrate metabolism [34]. Among individual olive phenolic compounds, luteolin exhibited the strongest α-amylase inhibitory activity, with an IC_50_ value of 36.09 ± 1.99 µg/mL, followed by hydroxytyrosol, oleuropein, tyrosol, and verbascoside, which showed lower or variable inhibitory effects depending on the extract matrix. Although these compounds demonstrated notable activity, their potency remained lower than that of acarbose (IC_50_ = 6.75 ± 0.15 µg/mL) under the reported conditions [35]. In contrast, methanolic olive stone extract (MSE) from *Olea europaea* L. var. Meski showed weaker α-amylase inhibition, with an IC_50_ value of 1.04 mg/mL (1040 µg/mL), while acarbose exhibited higher potency (IC_50_ = 0.39 mg/mL; 390 µg/mL). Despite the lower activity, these by-product extracts still demonstrate bioactive potential relevant to the regulation of postprandial glucose levels [36].

In vitro studies have consistently shown that *Punica granatum* L. extracts and their derivatives exhibit α-amylase inhibitory activity, although the potency varies depending on the extract type, plant part, and experimental conditions. The strongest inhibitory activity was reported by Badshah et al., who evaluated methanolic peel extracts from different pomegranate cultivars. The wild variety exhibited the highest potency (IC_50_ = 16.66 μg/mL), followed by the red (20.11 μg/mL) and white (25.37 μg/mL) varieties, indicating notable inhibitory activity across all tested samples [37]. Comparable inhibitory activity was reported by Royapuram Parthasarathy et al., who showed that strontium nanoparticles synthesized using *P. granatum* peel extract (PP-Sr NPs) exhibited an IC_50_ value of 38.07 μg/mL, which was close to that of acarbose (IC_50_ = 34.21 μg/mL) under the same experimental conditions [37]. Thapa et al. reported dose-dependent inhibition of α-amylase by methanolic P. granatum peel extract, with an IC_50_ value of 222.19 μg/mL. Under identical experimental conditions, acarbose exhibited stronger inhibitory activity (IC_50_ = 137.74 μg/mL), indicating lower potency of the plant extract. This effect was primarily attributed to ellagitannin-rich fractions capable of interacting with the enzyme’s active site [38]. In a comparative study by Kam et al., different *Punica granatum* L. components (flowers, peel, juice, and seeds) were evaluated. The methanolic flower extract exhibited the strongest inhibitory activity against porcine pancreatic α-amylase, followed by peel extract, whereas juice and seed extracts showed minimal inhibition. Among the identified compounds, punicalagin demonstrated inhibitory activity with an IC_50_ value of 140 ± 40 μg/mL, suggesting its contribution to the overall activity [39]. Lower inhibitory activity was reported by Laaraj et al., who showed that an acetone extract of *P. granatum* exhibited α-amylase inhibition with an IC_50_ value of 373.90 μg/mL [40]. Chukwuma et al. also reported the α-amylase inhibitory activity of an acetone extract of pomegranate fruit peel in vitro; however, no IC_50_ value was provided, and thus the inhibitory potency could not be quantitatively assessed [41].

α-Glucosidase is a membrane-bound enzyme located at the brush border of the small intestine and plays a key role in the final step of carbohydrate digestion and monosaccharide absorption. Direct comparisons between IC_50_ values of extracts (µg/mL) and isolated compounds (µM) should be interpreted with caution due to differences in molecular weight and assay conditions; therefore, cross-study comparisons should be considered indicative rather than absolute. Among olive-derived compounds, luteolin and oleuropein have been identified as strong α-glucosidase inhibitors. In the study by Haguet et al., luteolin exhibited a very low IC_50_ value of 0.4 µg/mL, while oleuropein showed an IC_50_ of 3.1 µg/mL, indicating high inhibitory potency and suggesting that specific olive phenolics are major contributors to carbohydrate metabolism modulation [42]. Similarly, Dekdouk et al. reported that luteolin, hydroxytyrosol, and oleuropein exhibited strong α-glucosidase inhibitory activity in vitro, with luteolin again showing the highest potency (IC_50_ = 14.12 ± 0.85 µg/mL). These findings indicate that isolated phenolic compounds may significantly contribute to the inhibitory activity observed in crude extracts [35]. Hydroxytyrosol and oleuropein also demonstrated dose-dependent α-glucosidase inhibition in vitro, with hydroxytyrosol showing stronger activity (IC_50_ = 150 ± 1.45 μM ≈ 23.12 µg/mL) compared to oleuropein (IC_50_ = 400 ± 1.25 μM ≈ 216.20 µg/mL). Both compounds exhibited higher inhibitory efficiency than acarbose under the same experimental conditions (IC_50_ = 820 ± 3.12 μM ≈ 529.39 µg/mL) [43]. Mansour et al. reported comparable α-glucosidase inhibitory activity across different olive cultivars (Picual, Tofahi, Shemlali) in vitro, with IC_50_ values ranging from 14.14 ± 0.41 to 14.61 ± 0.28 µg/mL, indicating relatively consistent bioactivity among genotypes [34]. In addition, a study by AlShaal et al. showed that olive leaf extracts exhibited 81.34% inhibition at 3.85 mg/mL, with an IC_50_ value of 340 ± 120 µg/mL, indicating moderate inhibitory activity compared to isolated olive-derived compounds in vitro [44]. Collectively, these findings indicate that phenolic constituents of *Olea europaea* L. contribute substantially to α-glucosidase inhibition, supporting their potential role in the regulation of postprandial glucose levels.

*Punica granatum* L. has attracted considerable attention as a source of bioactive compounds with potential antidiabetic properties, particularly through inhibition of α-glucosidase, a key enzyme involved in carbohydrate digestion. Multiple in vitro studies have demonstrated that different plant parts, including peel, flowers, and bark, as well as derived formulations, exhibit varying degrees of inhibitory activity. Royapuram Parthasarathy et al. reported that strontium nanoparticles synthesized using pomegranate peel extract (PP-Sr NPs) exerted a concentration-dependent inhibitory effect on α-glucosidase, reaching 76.17% inhibition at 160 μg/mL, with an IC_50_ value of 30.56 μg/mL. This activity was comparable to acarbose (IC_50_ = 29.42 μg/mL) under in vitro conditions and is likely associated with polyphenols and hydrolyzable tannins contributing to enzyme interaction via nanoparticle surface functionalization [37]. In addition to peel-derived systems, pomegranate flowers represent a rich source of structurally diverse phenolic compounds. Yuan et al. reported the isolation and identification of two new phenolic compounds, a coumarin, 7,8-dihydroxy-3-carboxymethylcoumarin-5-carboxylic acid featuring a novel carbon skeleton, and an unusual hydrolyzable tannin punicatannin C (80.34 ± 0.17 μg/mL) containing a norneolignan moiety, along with 10 known phenolics. All the isolates were evaluated for in vitro α-glucosidase (yeast and mammalian) inhibitory activities, indicating moderate to strong inhibition compared to acarbose (301.72 ± 18.93 μg/mL). The mechanism is generally attributed to hydrogen bonding and hydrophobic interactions with the enzyme, which may interfere with substrate binding and catalytic activity [45]. Bellesia et al. investigated ellagitannin-rich extracts using in vitro enzymatic assays combined with a simulated gastrointestinal digestion model. The extract exhibited strong α-glucosidase inhibition and weaker effects on α-amylase. Key compounds, including punicalagin (IC_50_ = 140.2 μmol/L ≈ 152.08 µg/mL), punicalin (IC_50_ = 191.4 μmol/L ≈ 149.74 µg/mL), and ellagic acid (IC_50_ = 380.9 μmol/L ≈ 115.10 µg/mL), were identified as major contributors. Kinetic analysis suggested a mixed-type inhibition mechanism, indicating interactions with both the free enzyme and the enzyme–substrate complex. Despite partial degradation during simulated digestion, the extract retained substantial inhibitory activity and reduced glucose release from starch-rich matrices in vitro [46]. Comparable findings were reported for pomegranate bark extracts. Kharchoufa et al. demonstrated that different solvent fractions exhibit distinct levels of α-glucosidase inhibition in vitro, with the acetone extract showing the strongest activity (IC_50_ = 96.19 μg/mL; 95.85% inhibition at 166 μg/mL), while aqueous, chloroform, and methanolic extracts exhibited higher IC_50_ values. The observed effects were associated with phenolic constituents such as ellagic acid, catechin, vanillin, and vanillic acid, suggesting their role in enzyme inhibition through interaction with the catalytic site [47]. An additional in vitro study by Çam et al. investigated phenolic-rich extracts from pomegranate peel. The optimized extract exhibited strong α-glucosidase inhibition, with an IC_50_ value of 5.56 ± 2.23 μg/mL. The inhibitory activity correlated with high total phenolic content (192.0 mg/g dry weight), indicating that polyphenols are the primary contributors. No significant differences were observed between optimized aqueous and conventional methanolic extracts, highlighting the efficiency of green extraction approaches [41]. Overall, these findings indicate that *Punica granatum*-derived extracts and compounds exhibit significant in vitro α-glucosidase inhibitory activity, with potency strongly influenced by plant part, extraction method, and chemical composition (Table 2). While nanoparticle-based formulations may enhance apparent efficacy, isolated compounds generally demonstrate moderate activity in the micromolar range. However, despite promising in vitro results, further in vivo and clinical studies are required to confirm their relevance in the management of postprandial hyperglycemia and to evaluate their potential as functional food ingredients or nutraceuticals.

The available in vitro evidence indicates that olive- and pomegranate-derived polyphenols possess considerable potential for the modulation of postprandial glucose metabolism through inhibition of α-amylase and α-glucosidase. Although the reported inhibitory potency varies substantially depending on plant part, extraction method, and phytochemical composition, several extracts and isolated compounds demonstrated activities comparable to or even exceeding those of conventional antidiabetic agents under experimental conditions.

Importantly, the observed effects appear to result not only from individual compounds such as luteolin, oleuropein, punicalagin, or ellagic acid, but also from synergistic interactions between multiple phenolic constituents present within complex plant matrices. This suggests that whole extracts or polyphenol-rich fractions may provide broader biological activity than isolated molecules alone. In addition, the simultaneous antioxidant and anti-inflammatory properties of these phytochemicals may further contribute to their beneficial effects in metabolic disorders, particularly considering the strong relationship between oxidative stress, chronic inflammation, and insulin resistance.

Nevertheless, interpretation of the current findings should be approached cautiously due to substantial methodological heterogeneity among studies, including differences in extraction procedures, enzyme sources, assay conditions, and the expression of inhibitory activity. Furthermore, the majority of available data originate from in vitro models, while information regarding in vivo efficacy, intestinal bioavailability, and long-term metabolic effects remains limited. Future studies should therefore focus on standardized experimental protocols and clinical validation in order to better define the therapeutic relevance of olive and pomegranate polyphenols in the management of type 2 diabetes and related metabolic disorders.

## 4. Neuroprotective Effects of Cholinesterase and Secretase Inhibition

Alzheimer’s disease is a progressive neurodegenerative disease that is characterized by a gradual decline in cognitive functions, such as memory, reasoning and language abilities. The pathogenesis of Alzheimer disease is not completely understood; however, the current model is primarily based on two key theories—the cholinergic and amyloid hypotheses. The cholinergic hypothesis is based on the reduced availability of acetylcholine (ACh) in the brain of patients, which is primarily associated with increased hydrolysis caused by acetylcholinesterase. Additionally, butyrylcholinesterase, also known as pseudocholinesterase, has been shown to contribute to the regulation of ACh levels in the brain, with its importance increasing in the advanced stages of the disease [48,49]. Butyrylcholinesterase performs a complementary function to AChE by hydrolyzing the ACh that diffuses from the synaptic cleft in the central nervous system and neuromuscular junction [50]. On the other hand, the amyloid hypothesis assumes that the accumulation and aggregation of amyloid-β (Aβ) peptides in the brain are an early and key step in the development of Alzheimer’s disease. Aβ is formed by sequential proteolytic cleavage of the amyloid precursor protein (APP) mediated by β-secretase and γ-secretase, whereby these secretases directly participate in their formation. A disturbed balance between Aβ production and elimination leads to its accumulation in the form of neurotoxic oligomers and extracellular plaques, resulting in synaptic dysfunction, neuroinflammation and progressive neuronal loss [51]. Considering the complex causes of Alzheimer’s disease, modern research increasingly points to the limitations of monotherapeutic, single-target approaches in treatment. Instead, emphasis is placed on the development of multi-target strategies that can simultaneously modulate multiple pathological processes, including oxidative stress, amyloid-β peptide aggregation, and cholinergic neurotransmission disorders [52].

Over decades, numerous AD treatment strategies have been developed. One of the most important strategies is the inhibition of acetylcholinesterase and butyrylcholinesterase [53]. Consequently, the search for effective and safe cholinesterase enzyme inhibitors from natural materials has expanded [54]. *Punica granatum* L. and *Olea europaea* L. have attracted attention due to their wide range of therapeutic properties, including the inhibition of key enzymes such as cholinesterase and secretase. These effects are mostly attributed to their rich phytochemical profile, particularly their high content of polyphenolic compounds, which are considered the main contributors to their bioactivity. Numerous studies have demonstrated the inhibitory effect of extracts and compounds isolated from these species against enzymes involved in neurodegenerative diseases [51,53,55].

Phenol-rich extracts of extra virgin olive oils were evaluated as multi-target ligands that inhibit multiple enzymes involved in Alzheimer’s and Parkinson’s diseases and major depressive disorder. Both extracts were able to simultaneously inhibit selected enzymes, which confirms their neuroprotective potential [56]. Olive leaves, as one of the most represented by-products in olive growing, are a priority subject of research for the purpose of their valorization. Studies conducted on olive leaf extracts from different growing regions, including Spain, Greece, Portugal, Italy, Turkey, Bosnia and Herzegovina, etc., indicate a significant inhibitory potential towards cholinesterases [57,58,59,60,61,62]. A recent investigation by Kaygisiz et al., 2024 [63], indicated that the inhibitory activity of olive leaf extracts toward AChE and BChE is influenced by harvest time and altitude. The samples harvested in winter and spring at higher altitudes exhibited enhanced inhibition of enzymes. The rich phenolic profile characterized with oleuropein and cynaroside as the main compounds showed significant correlation with observed neuroprotective potential [63]. Beyond the leaves, researchers have also focused on valorization of olive seeds and pomace. The results showed that ultrasound-assisted extracts from olive seeds have the ability to inhibit AChE and BChE. The authors emphasized that anti-neurodegenerative activity is significantly correlated with the total phenolic and flavonoid content, particularly with the presence of compounds such as tyrosol, rutin, luteolin-7-glucoside, oleuropein, and ligstroside [63]. A study of olive pomace juice enriched with hydroxytyrosol showed multiple neuroprotective activities. In the model of oxidative stress induced with H_2_O_2_ in HT22 cells, treatment with this juice significantly reduced the increased activity of BACE1 and AChE, and prevented the accumulation of amyloid fibrils. These results indicate that olive polyphenols such as hydroxytyrosol have a direct effect on suppressing the early causes that lead to the development of Alzheimer’s disease [64]. Phenolic compounds isolated from *Olea europaea* L. have been explored as a potential source for the development of new AChE inhibitors. For instance, tyrosol has been used for the synthesis of triazole analogs. Among 15 synthesized compounds, 7-({1-[2-(4-hydroxyphenyl)ethyl]-1*H*-1,2,3-triazol-4-yl}methoxy)-4-methyl-2*H*-chromen-2-one showed the highest inhibitory activity, with an IC_50_ value of 14.66 µmol/L [65]. Innovative approaches to the creation of functional natural preparations have led to research into the valorization of olive oil by enriching it with edible freeze-dried insects. Earlier studies of these freeze-dried insects have shown that they are a good source of vitamins B and C, as well as phenolic compounds. The incorporation of freeze-dried biomass into olive oil not only contributed to the enrichment of the nutritional profile, but also led to a significant increase in the inhibitory activity against acetylcholinesterase. This integrative approach indicates the potential of synergistic products as neuroprotective agents [66].

A similar neuroprotective potential, which includes inhibition of AChE and BChE, as well as modulation of the amyloid cascade, is also shown by various pomegranate extracts. The specific profile of ellagitannin makes pomegranate a source of multi-target agents. The therapeutic efficacy of pomegranate is closely linked to metabolic conversion of ellagitannins into urolithins. A recent study (Noshadi et al., 2020) [67] has demonstrated that urolithins, along with their methyl ether derivates, exhibit inhibitory activity against cholinesterases and several other enzymes related to neurodegenerative diseases. In the same study, molecular docking analysis confirms their potential as multi-target agents, showing favorable interactions within the active sites of the mentioned enzymes [67]. In the study of Kwak et al. (2005) [49], two bioactive compounds isolated from the pomegranate husk, punicalagin and ellagic acid, showed inhibitory activity against several enzymes, including β-secretase, α-secretase, elastase, chymotrypsin and trypsin. Ellagic acid and punicalagin had IC_50_ values of 3.9 × 10^−6^ M and 4.1 × 10^−7^ M, respectively. The obtained results indicated significant and specific inhibitory potential toward BACE1 when compared to the other investigated enzymes, suggesting their impact as targeted neuroprotective compounds [49]. Similarly, methanolic and ethanolic extracts of *Punica granatum* L. exhibited potent acetylcholinesterase inhibition comparable to tacrine, the first FDA-approved drug for AD treatment. According to UPLC-MS/MS, ellagic acid was identified as the major compound, which suggests a strong correlation between its high concentration and AChE inhibition [68]. The potential of pomegranate industry by-products was examined in a study of pomegranate seed extract. Detailed phytochemical analysis, performed by LC-MS/MS and GC-MS analysis, identified ellagic acid, gallic acid, 2-oxatricyclo[4.3.1.0(3,8)]decane, 2-heptenal, 2-propyl- and 1*H*-indene-1-one as the main constituents. The extract showed high anticholinesterase activity, inhibiting AChE by up to 67.8% and BChE by up to 79.8%. The neuroprotective potential was further confirmed by molecular docking and drug-likeness testing of the main constituents (Table 3). Specifically, the major compound showed favorable log *p* and TPSA values, suggesting high bioavailability and potential to cross the blood–brain barrier [69].

The presented findings suggest that olive and pomegranate polyphenols may exert neuroprotective effects through simultaneous modulation of several pathological mechanisms involved in Alzheimer’s disease progression. Unlike conventional single-target approaches, many of the investigated compounds demonstrated the ability to inhibit cholinesterases while also affecting β-secretase activity, oxidative stress, and amyloid-related pathways. Such multi-target activity is particularly important considering the complex and multifactorial nature of neurodegenerative disorders. An additional advantage of these phytochemicals lies in their natural origin and long-term presence within the Mediterranean diet, which may support better tolerability and broader preventive applicability. Furthermore, the observed correlations between phenolic composition and enzyme inhibition indicate that synergistic interactions between multiple compounds likely contribute to the overall biological activity, rather than indicating the effect of a single dominant molecule. Nevertheless, despite highly promising in vitro evidence, translation into clinical relevance remains challenging due to limitations associated with bioavailability, blood–brain barrier penetration, and metabolic transformation of polyphenols after ingestion. Therefore, future investigations should focus on integrating enzymatic studies with pharmacokinetic and in vivo approaches in order to better define the true therapeutic potential of olive- and pomegranate-derived compounds in neurodegenerative disease prevention and treatment. A major limitation is the limited information regarding the ability of many bioactive compounds to cross the blood–brain barrier (BBB) in sufficient concentrations. While some low-molecular-weight phenolics and their metabolites may exhibit improved bioavailability and BBB permeability, evidence remains incomplete for many of the compounds discussed in this review. Consequently, enzyme inhibition observed in vitro should not be interpreted as direct evidence of neuroprotective efficacy in vivo. Additional pharmacokinetic studies, BBB permeability assessments, and animal or clinical investigations are required to clarify the relevance of these findings for neurodegenerative disorders.

AChE inhibition has been investigated more extensively and is commonly used as an indicator of potential anti-Alzheimer activity. However, increasing evidence indicates that BChE activity becomes more important during the progression of Alzheimer’s disease, making dual inhibition a potentially advantageous strategy. Several olive- and pomegranate-derived phenolics exhibit inhibitory activity against both enzymes, although the degree of inhibition was not always comparable. Differences in enzyme structure, substrate specificity, and binding interactions may contribute to the observed variability. Nevertheless, direct quantitative comparisons between studies remain challenging because of differences in assay conditions, enzyme sources, and reporting methods.

Therefore, compounds demonstrating balanced inhibition of both AChE and BChE may possess greater therapeutic potential than compounds acting predominantly on a single target. Future investigations should incorporate standardized experimental conditions and comparative kinetic analyses to better define enzyme selectivity and therapeutic relevance.

## 5. Inhibition of Drug-Metabolizing Enzymes: The Cytochrome P450 System

Oleuropein, a polyphenol abundant in olives, has been shown to be an inhibitor of the CYP3A enzyme, inhibiting androstenedione β-hydroxylase by 42% [71]. Oleuropein also inhibited CYP1A2-mediated 7-methoxyresorufin O-deethylation by 24%, while it did not show an inhibitory effect on CYP2E1-mediated chlorzoxazone 6-hydroxylation [72]. In a study by Rodríguez-Morató et al. (2017) [73], it was demonstrated that the CYP2A6 and CYP2D6 enzymes participate in the biotransformation of tyrosol into hydroxytyrosol, a polyphenol with many beneficial effects on human health [73]. Pomegranate juice has shown an in vitro inhibitory effect on the carbamazepine 10,11-epoxidase activity of the CYP3A enzyme. Hidaka et al. (2005) demonstrated a very strong inhibition of human CYP3A carbamazepine 10,11-epoxidase activity, while Farkas et al. (2007) reported that the IC_50_ for the inhibition of triazolam α-hydroxylation, metabolized by the CYP3A enzyme, was 0.61% for pomegranate juice [74,75]. Usia et al. (2006) [76] investigated the inhibitory effects of methanolic extracts from 30 plant samples on CYP3A4 and CYP2D6 enzymes. The inhibitory effect of the MeOH-soluble fraction of pomegranate on CYP2D6 was 98.1%, the highest among all tested plants, while its inhibitory effect on the CYP3A4 enzyme exceeded 70%. In contrast, ethanolic extracts of pomegranate showed an inhibitory effect of less than 10% on both enzymes [76] (Table 4).

## 6. Multi-Target Therapeutic Potential and Translational Perspectives

The findings summarized in this review indicate that bioactive compounds derived from *Olea europaea* L. and *Punica granatum* L. should not be considered as single-purpose phytochemicals, but rather as multi-target modulators capable of simultaneously influencing several pathological pathways associated with chronic diseases. This characteristic is particularly important in the context of multifactorial disorders such as metabolic syndrome, neurodegenerative diseases, and chronic inflammatory conditions, where multiple biochemical mechanisms contribute to disease progression.

One of the most significant observations emerging from the reviewed studies is the ability of olive and pomegranate polyphenols to interact with interconnected enzyme systems. For example, compounds such as oleuropein, hydroxytyrosol, punicalagin, and ellagic acid exhibit inhibitory activity against inflammatory enzymes (COX and LOX) and carbohydrate-digesting enzymes (α-amylase and α-glucosidase), as well as neurodegeneration-related enzymes including acetylcholinesterase, butyrylcholinesterase, and β-secretase. Such broad-spectrum activity suggests that these phytochemicals may provide synergistic therapeutic effects extending beyond isolated pharmacological targets.

The concept of multi-target enzyme modulation is increasingly recognized as a promising strategy in modern pharmacology because many chronic diseases share common pathological mechanisms, including oxidative stress, chronic low-grade inflammation, mitochondrial dysfunction, and impaired cellular signaling. In this context, olive- and pomegranate-derived polyphenols may represent valuable candidates for the development of functional foods, nutraceuticals, or adjunct therapeutic agents. Their natural origin and long-term presence within the Mediterranean diet further support their translational relevance.

However, despite promising in vitro findings, several limitations must be acknowledged. Most available studies rely on isolated enzyme assays or simplified cellular models, which may not fully reflect physiological conditions in vivo. In addition, the bioavailability, metabolic transformation, and tissue distribution of many polyphenols remain insufficiently understood. Compounds such as punicalagin and oleuropein undergo extensive metabolism after ingestion, producing derivatives that may differ substantially in biological activity from their parent molecules. Furthermore, methodological heterogeneity between studies, including differences in extraction procedures, assay conditions, and reporting units, complicates direct comparisons of inhibitory potency.

Another important consideration is the interaction of these phytochemicals with cytochrome P450 enzymes. Although CYP inhibition may enhance the therapeutic activity of certain compounds, it also raises concerns regarding potential herb–drug interactions, especially in patients receiving long-term pharmacotherapy. Therefore, future research should focus not only on confirming efficacy in vivo and in clinical studies, but also on evaluating pharmacokinetic safety and interaction profiles.

Overall, the current evidence supports the view that olive and pomegranate polyphenols act as biologically versatile compounds with considerable pharmaceutical and nutraceutical potential. Future investigations integrating enzymology, metabolomics, molecular docking, and clinical validation will be essential for translating these promising in vitro findings into evidence-based therapeutic applications.

The available evidence suggests that several phenolic compounds, including punicalagin, ellagic acid, oleuropein, and related metabolites, can affect CYP450 activity under experimental conditions. Nevertheless, the magnitude of these interactions in vivo remains uncertain because the enzyme inhibition observed in vitro may not accurately reflect concentrations achieved in human tissues following dietary consumption or supplementation. CYP450 inhibition should be interpreted within a benefit–risk framework. While enzyme modulation may contribute to multi-target therapeutic effects, it may simultaneously increase the risk of herb–drug interactions, particularly in individuals receiving medications with narrow therapeutic indices. Further pharmacokinetic, toxicological, and clinical studies are therefore required to determine the clinical significance of these observations.

## 7. Bioavailability and Metabolic Transformation of Bioactive Compounds

Although numerous studies have demonstrated potent enzyme inhibition by olive and pomegranate polyphenols under in vitro conditions, their biological effects in vivo are strongly influenced by bioavailability, intestinal absorption, and metabolic transformation. In pomegranate, ellagitannins such as punicalagin are extensively metabolized by the gut microbiota to urolithins, particularly urolithin A, which may represent the actual bioactive forms reaching systemic circulation. Consequently, enzyme inhibitory effects observed for parent compounds in vitro may not fully reflect the physiological situation.

Similarly, olive phenolics undergo extensive metabolism involving glucuronidation, sulfation, and methylation. Therefore, circulating metabolites rather than the native compounds may contribute substantially to the biological effects observed in vivo. Future studies should evaluate enzyme inhibition using physiologically relevant metabolites and concentrations.

## 8. Methods

### 8.1. Literature Search Strategy

The present review was conducted following the Preferred Reporting Items for Systematic Reviews and Meta-Analyses (PRISMA) guidelines. A comprehensive literature search was performed using the PubMed and Web of Science databases to identify studies investigating the enzyme inhibitory activities of bioactive olive (*Olea europaea* L.) and pomegranate (*Punica granatum* L.) compounds and extracts. The search strategy combined relevant keywords and Boolean operators, including: (“*Olea europaea*” OR olive OR olive phenolics OR oleuropein OR hydroxytyrosol) AND (“*Punica granatum*” OR pomegranate OR punicalagin OR ellagic acid) AND (“enzyme inhibition” OR α-glucosidase OR α-amylase OR cholinesterase OR acetylcholinesterase OR butyrylcholinesterase OR cyclooxygenase OR lipoxygenase OR CYP450). Additional searches were conducted using combinations of individual enzyme targets and specific phytochemicals where necessary. Only studies published in English were considered. This systematic review was created by searching the PubMed and Web of Science databases for papers published with the keywords “*Olea europaea* L., *Punica granatum* L.; glucosidase, amylase, COX, LOX, acetylcholinesterase, butyrylcholinesterase, secretase, CYP; inhibition; inhibitory” for the period up to 15 April 2026. The literature search was conducted in PubMed and Web of Science using the following Boolean combinations: (“*Olea europaea*” OR olive) AND (“*Punica granatum*” OR pomegranate) AND (“enzyme inhibition” OR “α-glucosidase” OR “α-amylase” OR AChE OR BChE OR COX OR LOX OR CYP450). Additional filters included in vitro studies, English language, and original research articles. The search resulted in 1000 papers: 321 from the PubMed database and 679 from the Web of Science database. After removing duplicates using Zotero software (version 9.0.1 (64-bit), Corporation for Digital Scholarship, Vienna, VA, USA), 499 papers remained. PubMed and Web of Science were selected because they provide broad coverage of the biomedical, pharmacological, and life-science literature and are widely used in systematic reviews of bioactive compounds and enzyme inhibition. Scopus was not included, which may have introduced database selection bias. Meta-analysis was not performed because enzyme sources, substrates, extract types, concentrations, IC_50_ units, and assay conditions were highly heterogeneous. After initial analysis and abstract screening, a total of 177 full-text articles were assessed for eligibility, and 57 papers were selected for the study. Exclusion criteria were irrelevance, non-related enzymes, different plant species, or non-in vitro design. The primary outcomes we sought were quantitative measures of enzyme inhibition, specifically half-maximal inhibitory concentration (IC_50_) values, percent enzyme inhibition, and relevant enzyme kinetic parameters. We also recorded the source plant, specific plant parts used, type of extract administered, and isolated phenolic compounds tested. For the synthesis, eligible studies were grouped according to the physiological role of their target enzymes: inflammatory pathways, carbohydrate digestion, neuroprotection, and inhibition of the drug-metabolizing cytochrome P450 enzymes. Data are presented in tables to visually illustrate the findings.

### 8.2. Quality Assessment and Risk of Bias

Given the predominance of in vitro studies and the methodological heterogeneity among investigations, a formal clinical risk-of-bias tool was not applicable. Nevertheless, study quality was assessed according to the following criteria:

- Clear identification of tested compounds or extracts;
- Description of extraction and analytical procedures;
- Use of appropriate positive controls;
- Reporting of quantitative inhibition parameters (IC_50_, Ki, or percentage inhibition);
- Replication and statistical analysis of experiments.

The results of this assessment were considered during data interpretation and discussion of study limitations. The methodological appraisal of all included studies is provided in Supplementary Table S1.

### 8.3. Data Synthesis

Due to substantial heterogeneity among studies regarding enzyme sources, assay conditions, substrates, extraction procedures, concentrations, and outcome reporting units, a quantitative meta-analysis was not considered appropriate. Therefore, findings were synthesized narratively, with emphasis on comparative evaluation of enzyme targets, bioactive compounds, extraction methods, and translational relevance.

A direct comparison of inhibitory potency across studies should be interpreted with caution. Considerable methodological heterogeneity exists regarding enzyme origin, substrate type, incubation conditions, extract preparation, purification procedures, and concentration ranges. Furthermore, IC_50_ values were reported in different units and experimental systems, limiting direct quantitative comparisons. Consequently, statements suggesting that a compound or extract is “stronger than acarbose” should be considered only within the specific experimental context in which the comparison was performed rather than as a universal conclusion.

## 9. Conclusions

Olive and pomegranate contain polyphenolic compounds that have proven beneficial effects on human health. Both plants have shown inhibitory effects on not only a single enzyme or group of enzymes, but on several different enzymes with various roles in the human body, opening the possibility of using olive and pomegranate as new auxiliary therapeutic preparations and dietary supplements that can help in the prevention and treatment of neurodegenerative diseases, inflammatory diseases, and metabolic diseases. It is important to note the possible interactions with drugs due to the inhibition of certain CYP enzymes. Of course, additional in vivo and clinical studies are needed to expand the understanding of the real impact of polyphenols from these two plants on human health.

## Figures and Tables

**Table 1:** Inhibitory effects of olive and pomegranate extracts/compounds on inflammatory enzymes.

Plant Source	Extracts/Compounds	Target Enzyme	Main Effect Observed	Reference
*Olea europaea* L.	Oleocanthal	COX-1, COX-2	Strong inhibition of prostaglandin synthesis; ibuprofen-like activity	[21,22,23,24]
*Olea europaea* L.	Hydroxytyrosol	5-LOX	Reduced leukotriene B4 production	[29,30]
*Olea europaea* L.	Oleuropein	5-LOX	Inhibition of inflammatory leukotriene pathway	[24,29]
*Punica granatum* L.	Punicalagin	COX-2	Suppression of COX-2 expression and inflammatory signaling	[25,26,27,28]
*Punica granatum* L.	Urolithin A	COX-2	Reduced prostaglandin production	[26]
*Punica granatum* L.	Polyphenol-rich extracts	LOX-related pathways	Reduction in leukotriene formation and oxidative stress	[27,31,32]

**Table 2:** In vitro inhibitory activity of olive and pomegranate extracts/compounds against carbohydrate-digesting enzymes.

Plant Source	Extracts/Compounds	Target Enzyme	IC_50_ Value	Key Findings	Reference
*Olea europaea* L.	Aqueous olive leaf extract	α-Amylase	0.75 µg/mL	Stronger activity than acarbose	[33,34]
*Olea europaea* L.	Luteolin	α-Amylase	36.09 ± 1.99 µg/mL	Most potent olive phenolic	[35]
*Olea europaea* L.	Luteolin	α-Glucosidase	0.4 µg/mL	Very strong inhibitory activity	[42]
*Olea europaea* L	Oleuropein	α-Glucosidase	3.1 µg/mL	Potent inhibitor	[42]
*Punica granatum* L.	Methanolic peel extract	α-Amylase	16.66 µg/mL	Strong inhibition in wild cultivar	[37]
*Punica granatum* L.	PP-Sr nanoparticles	α-Glucosidase	30.56 µg/mL	Comparable to acarbose	[37]
*Punica granatum* L.	Punicalagin	α-Glucosidase	152.08 µg/mL	Mixed-type inhibition	[46]
*Punica granatum* L.	Optimized peel extract	α-Glucosidase	5.56 ± 2.23 µg/mL	High phenolic-related activity	[41]

**Table 3:** Neuroprotective enzyme inhibition by olive and pomegranate extracts/compounds.

Plant Source	Extracts/Compounds	Target Enzyme	Key Findings	Reference
*Olea europaea* L.	Olive leaf extracts	AChE, BChE	Significant cholinesterase inhibition	[57,58,59,60,61,62]
*Olea europaea* L.	Hydroxytyrosol-enriched olive pomace juice	AChE, BACE1	Reduced amyloid fibril accumulation	[64]
*Olea europaea* L.	Tyrosol triazole analog	AChE	IC_50_ = 14.66 µmol/L	[65]
*Punica granatum* L.	Urolithins	AChE, BChE	Multi-target neuroprotective activity	[70]
*Punica granatum* L.	Punicalagin	BACE1	Strong β-secretase inhibition	[48]
*Punica granatum* L.	Ellagic acid	BACE1	Potent inhibition of amyloid pathway	[48]
*Punica granatum* L.	Seed extract	AChE, BChE	Up to 79.8% inhibition	[68]

**Table 4:** Effects of olive and pomegranate extracts/compounds on cytochrome P450 enzymes.

Plant Source	Extracts/Compounds	Target Enzyme	Key Findings	Reference
*Olea europaea* L.	Oleuropein	CYP3A	42% inhibition of androstenedione β-hydroxylase	[69]
*Olea europaea* L.	Oleuropein	CYP1A2	24% inhibition of 7-methoxyresorufin O-deethylation	[71]
*Olea europaea* L.	Tyrosol	CYP2A6, CYP2D6	Biotransformation into hydroxytyrosol	[72]
*Punica granatum* L.	Pomegranate juice	CYP3A	Strong inhibition of carbamazepine metabolism	[73]
*Punica granatum* L.	Pomegranate juice	CYP3A	IC_50_ = 0.61% for triazolam α-hydroxylation	[74]
*Punica granatum* L.	Methanolic extract	CYP2D6	98.1% inhibition	[76]
*Punica granatum* L.	Methanolic extract	CYP3A4	>70% inhibition	[76]

## Data Availability

No new data were created or analyzed in this study. Data sharing is not applicable to this article.

## Supplementary Materials

The following supporting information can be downloaded at: https://www.mdpi.com/article/10.3390/molecules31122134/s1, Supplementary Table S1. Methodological appraisal of evidence included in the review; PRISMA 2020 checklist.

## Abbreviations

The following abbreviations are used in this manuscript:

Aβ	Amyloid-β
ACh	Acetylcholine
AChE	Acetylcholinesterase
AD	Alzheimer’s disease
APP	Amyloid precursor protein
BACE1	β-secretase
BChE	Butyrylcholinesterase
COX	Cyclooxygenase
CYP	Cytochrome P450
LOX	Lipoxygenase
MSE	Methanolic olive stone extract
PP-Sr NPs	Strontium nanoparticles synthesized using pomegranate peel extract
UOLE	Aqueous olive leaf extract
