# Supplementary material for: Enzyme Inhibition by Bioactive Compounds from Olive (Olea europaea L.) and Pomegranate (Punica granatum L.): Systematic Review of In Vitro Studies
Source: Molecules. 2026 Jun 17;31(12):2134. doi: 10.3390/molecules31122134 (PMC13306215; doi:10.3390/molecules31122134)
Supplement: Supplementary file 1 [file molecules-31-02134-s001.zip › Supplementary Table S1.pdf]

Supplementary Table S1. Methodological appraisal of evidence included in the review

Evidence domain	No of studies	Main strenghts	Main limitations	Overall confidence
COX/LOX inhibition	6	Identification of active compounds (oleocanthal, hydroxytyrosol, punicalagin, ellagic acid); quantitative inhibition reported in most studies	Limited kinetic characterization; scarce Ki values; mostly in vitro models	Moderate
α-Amylase and α-glucosidase inhibition	8	Frequent use of acarbose as positive control; IC_{50} values commonly reported	Considerable variability in assay conditions and reporting units; limited comparability among studies	Moderate
Cholinesterase inhibition (AChE/BChE)	7	Multiple plant extracts and isolated compounds evaluated; reproducible inhibitory trends	Few mechanistic studies; limited BBB relevance	Moderate
BACE1 and neuroprotective targets	3	Mechanistic relevance for Alzheimer's disease	Limited number of studies; predominantly screening-level evidence	Low–Moderate
CYP450 inhibition	7	Clinically relevant targets investigated; consistent evidence of enzyme interaction potential	Lack of in vivo confirmation; uncertainty regarding actual exposure levels	Moderate
Nanoparticle formulations	2	Enhanced enzyme inhibition compared with corresponding crude extracts	Limited translational evidence; formulation-dependent effects	Low
Isolated olive phenolics	Multiple studies	Better chemical characterization and mechanistic interpretation	Bioavailability issues rarely addressed	Moderate

Isolated pomegranate phenolics and metabolites	Multiple studies	Strong enzyme inhibition frequently observed; metabolite relevance emerging	Limited data on metabolite concentrations achieved in vivo	Moderate

The evidence was appraised qualitatively because the included studies exhibited substantial methodological heterogeneity regarding extraction procedures, enzyme sources, assay conditions, outcome reporting, and experimental design. Consequently, a formal quantitative risk-of-bias assessment was not considered appropriate. Instead, the studies were critically evaluated according to the consistency of findings, methodological transparency, use of positive controls, and availability of quantitative inhibition data.
